# Hierarchical Residual Attribution with Supervised Channel Shortlisting for Sensor-Local and Process Fault Diagnosis in Multivariate Sensor Time Series

**DOI:** 10.3390/s26154986

**Published:** 2026-08-06

**Authors:** Yuchen Wang, Xinran Lu, Jun Wang

**Affiliations:** College of Electronics and Information Engineering, Sichuan University, Chengdu 610065, China; wangyuchen7@stu.scu.edu.cn (Y.W.); luxinran@stu.scu.edu.cn (X.L.)

**Keywords:** sensor fault diagnosis, residual attribution, multivariate time series, injected perturbations, source-level macro-F1, affected-channel shortlisting, IoT sensing

## Abstract

Binary anomaly alarms are often insufficient for sensor systems because maintenance actions depend on whether abnormal behavior is localized to a sensor channel or reflects a process-level event. This paper proposes Hierarchical Residual Attribution with supervised channel shortlisting (HRA-SL), a diagnostic framework for multivariate sensor windows under a controlled injected-perturbation protocol. HRA fits calibration-based cross-channel consistency residuals for source-level diagnosis; HRA + spectral is the main source-diagnosis configuration; and HRA-SL adds supervised channel-candidate shortlisting for affected-channel localization. The evaluation uses synthetic, UCI HAR, and UCI Air Quality base signals with split-before-injection protocols over five seeds and five severity levels. HRA + spectral improves source-level macro-F1 over statistical, spectral, statistical + spectral, tuned TCN-AE, tuned USAD-like AE, and GRU-AE reconstruction-feature baselines, reaching 0.780±0.028, 0.655±0.017, and 0.554±0.029, respectively. Ablations show that HRA-Core carries most source-level signal, while residual-attribution features support channel ranking and diagnostic interpretation. The fixed HGB HRA-SL localizer reaches top-1 affected-channel localization of 0.916, 0.714, and 0.715. Under matched target-channel supervision, HRA-SL is practically tied with AE-only HGB on synthetic data (0.916 versus 0.918) and UCI HAR (0.714 versus 0.716), and is higher on Air Quality (0.715 versus 0.652). It also improves over the strongest unsupervised tuned reconstruction ranking baseline by 0.127, 0.357, and 0.048 top-1 under different supervision assumptions. The study establishes a controlled-injection benchmark for reproducible sensor source diagnosis and affected-channel shortlisting on public base signals, providing pre-deployment evidence that can be extended to field-fault studies with maintenance records.

## 1. Introduction

Sensor anomaly detection is commonly evaluated as a binary task: a multivariate window is either normal or anomalous. For sensor-system operation, that answer is often too coarse. A sensor-local fault such as drift, dropout, clipping, a time lag, or a noise-floor change points toward maintenance, recalibration, or channel replacement. A process fault instead suggests intervention in the monitored physical or environmental process. The source of the anomaly therefore determines the response beyond the presence of an alarm.

This paper studies source-level diagnosis for multivariate sensor time series using a reproducible injected-perturbation protocol. The task separates three decisions: detecting whether a window is faulty, diagnosing whether the fault is sensor-local or process-level, and shortlisting likely affected channels for sensor-local faults.

**Figure 1:**
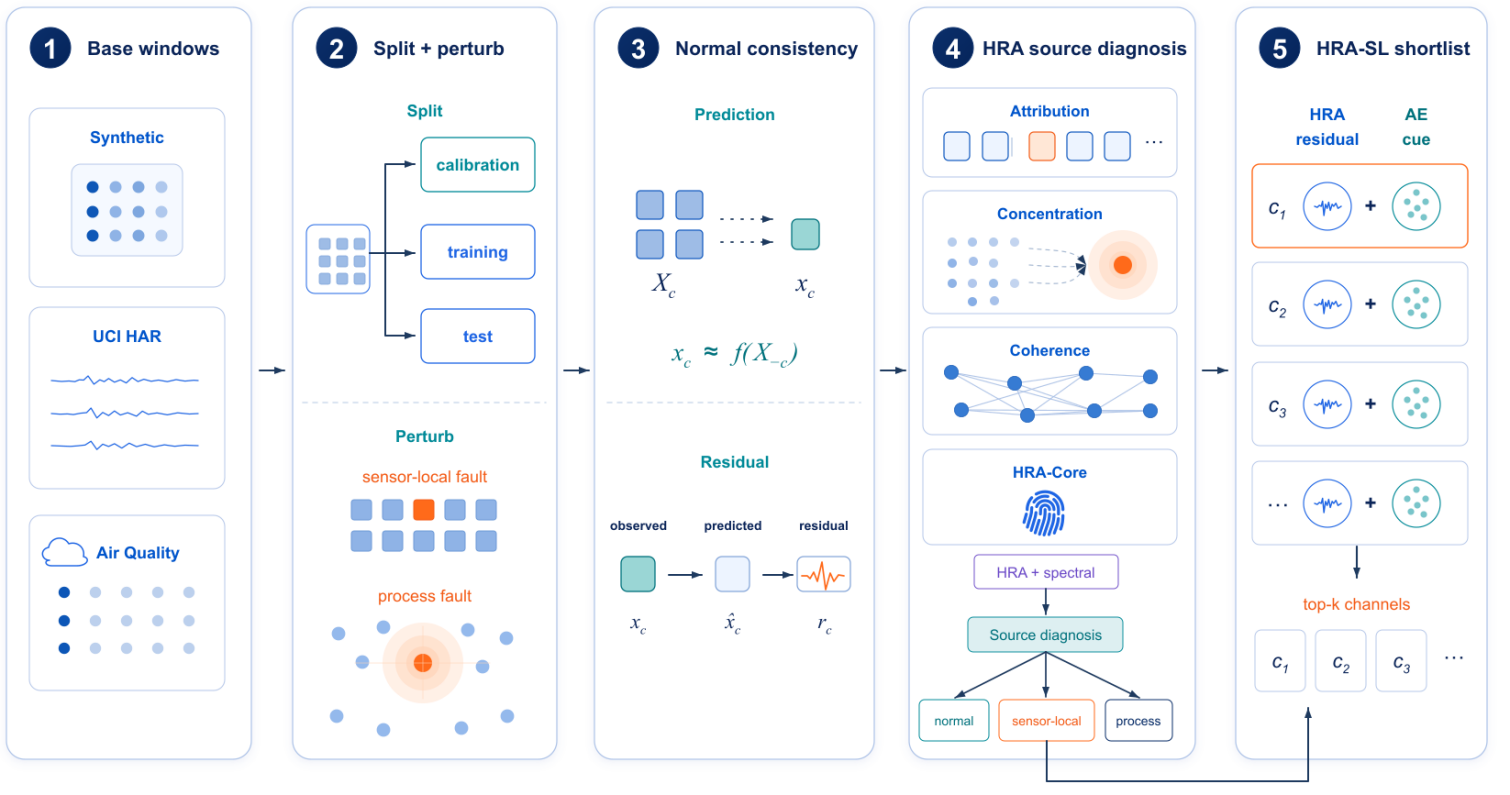
HRA-SL diagnostic workflow. Controlled perturbations are injected into synthetic or public base sensor windows. Calibration-fitted cross-channel consistency models produce HRA-Core and residual-attribution features for source-level diagnosis. For sensor-local training windows, target-channel labels supervise a channel-candidate shortlisting model, and the test split provides localization metrics.

We propose Hierarchical Residual Attribution with supervised channel shortlisting (HRA-SL), a diagnostic framework for this task. The framework separates three modules: HRA is the source-level residual-diagnosis component, HRA + spectral is the main source-diagnosis configuration, and HRA-SL is the supervised shortlisting extension for affected-channel localization. The HRA component learns normal cross-channel consistency from calibration windows, computes residual attribution scores for each channel, summarizes residual concentration and residual coherence, and concatenates these features with a calibration-fitted HRA-Core diagnostic signature block. A diagnostic probe separates normal, sensor-local, and process-fault windows. For the localization step, HRA-SL treats each channel in a sensor-local training window as a candidate and learns a supervised shortlisting model from training-split target-channel labels. Figure 1 summarizes the workflow.

The validation scope is deliberate: controlled injected perturbations provide known source and channel labels on public base signals, enabling repeatable diagnosis tests before field-fault studies with maintenance records and repair outcomes.

The main contributions are:

1. We introduce HRA, a calibration-fitted residual source-diagnosis component that converts cross-channel consistency errors into magnitude, concentration, and coherence evidence for normal, sensor-local, and process-fault decisions.
2. We extend HRA to HRA-SL, a supervised affected-channel shortlisting module that combines HRA channel cues with autoencoder reconstruction-energy cues in a matched channel-candidate ranking protocol.
3. We evaluate the method over three datasets, five seeds, and five severity levels with split-before-injection controls, reporting source-diagnosis gains, matched-supervision shortlisting comparisons, and per-run reproducibility artifacts.

## 2. Related Work

### 2.1. Multivariate Time-Series Anomaly Detection

Multivariate time-series anomaly detection has been studied with reconstruction, forecasting, recurrent, graph, contrastive, and transformer models. USAD [1] uses adversarially trained autoencoders for multivariate anomaly detection; OmniAnomaly [2] uses stochastic recurrent modeling; GDN [3] learns graph structure among sensors; TranAD [4] applies transformer networks to multivariate anomaly detection; and Anomaly Transformer [5] uses association discrepancy for time-series anomaly scoring. Interpretable multivariate anomaly systems include hierarchical inter-metric temporal embedding [6], Microsoft time-series anomaly detection service [7], TadGAN [8], MAD-GAN [9], STAD-GAN [10], DCdetector [11], Deep Isolation Forest [12], graph-transformer anomaly detection for IoT [13], and recent Granger-causal root-cause analysis [14]. Reviews summarize the breadth of time-series anomaly-detection methods [15,16], and benchmark studies show that reported performance is sensitive to evaluation protocol, thresholding, and dataset choice [17,18]. IoT anomaly-detection surveys similarly highlight time-series anomaly-detection challenges and deployment constraints [19].

These methods primarily output anomaly scores, reconstruction errors, or forecasting errors. Fewer studies jointly evaluate source-level diagnosis and affected-channel shortlisting under a split-before-injection protocol. HRA-SL instead evaluates whether residual structure can support both decisions after a window becomes abnormal.

### 2.2. Residual-Based Fault Diagnosis

Residuals are central in classical model-based and data-driven fault diagnosis. Model-based fault-diagnosis reviews [20] describe residual generation, evaluation, and isolation as core ideas for diagnosing deviations from expected behavior, while process-monitoring reviews [21] cover data-driven monitoring and diagnosis more broadly. Sensor-network fault taxonomies [22] catalog common data fault types, including outliers, stuck-at readings, noise, and calibration errors. Recent work on separating sensor anomalies from process anomalies [23] is especially close to our source-level diagnosis goal. Industrial transfer-learning surveys [24], domain feature decoupling for rotating-machinery fault diagnosis [25], joint collaborative adaptation for rolling-bearing diagnosis [26], and time-frequency pretraining for rotating machinery [27] illustrate the growing role of learned representations in sensor diagnostics. The rotating-machinery studies address supervised or transfer diagnosis under unseen operating conditions, class imbalance, or variable operating regimes in vibration-like machinery signals. HRA-SL instead evaluates source-level sensor-local/process diagnosis and affected-channel shortlisting in multivariate sensor windows, where the output is not a machine-condition class alone but also a ranked channel candidate list.

Acoustic-emission localization methods, including deep residual learning for steel-concrete composite slabs [28] and enhanced guided-wave phased-array localization [29], also localize physical sources from sensor signals, but their acoustic modality, geometry assumptions, and spatial labels differ from source-level sensor-local/process diagnosis and channel-candidate shortlisting.

HRA builds on this residual-diagnosis lineage by converting calibration-fitted residuals into channel attribution, concentration, and coherence features for source diagnosis and affected-channel shortlisting. The remaining gap is a reproducible task protocol that tests whether such residual evidence separates sensor-local faults from process-level events while also ranking affected channels.

### 2.3. Reproducible Sensor Benchmarks

Public benchmark signals support reproducible evaluation. UCI HAR [30] provides six inertial smartphone channels from body acceleration and gyroscope signals. UCI Air Quality provides environmental gas and concentration channels from an urban pollution monitoring scenario. Industrial process benchmarks such as SWaT [31] further motivate reproducible sensor-system evaluation. What is less common is using public base signals to create controlled source labels while preserving train/test separation before perturbation injection. We use public signals as base windows under the split and perturbation protocol described below.

### 2.4. Time-Series Representations and Probes

Modern time-series representation learning has expanded beyond anomaly scoring. TS2Vec [32], CoST [33], time-frequency contrastive pre-training [34], TimesURL [35], Time2Feat [36], and ShapeFormer [37] show that time-series windows can be represented through contrastive, interpretable, or shapelet-like feature spaces. Forecasting-oriented architectures such as Informer [38], PatchTST [39], TimesNet [40], Chronos [41], MOMENT [42], and Timer [43] provide additional context for time-series feature transfer. For supervised probes, LSTM-FCN [44] and ROCKET [45] motivate feature-and-probe comparisons. These works motivate strong feature baselines, but they do not directly answer how interpretable residual signatures, spectral features, and supervised channel-candidate probes behave in a source-level diagnostic workflow.

## 3. Materials and Methods

### 3.1. Problem Formulation

Let $X\in R^{T\times C}$ denote a multivariate sensor window with length *T* and *C* channels. The source-level target is

y∈{normal,sensor-local fault,process fault}.

For sensor-local faults, the perturbation generator records a target channel $c^{★}$. Source-level diagnosis is trained from window features and source labels. In the supervised shortlisting setting, $c^{★}$ labels positive channel candidates in the supervised-training split and defines localization metrics on the test split.

The primary endpoint is source-level macro-F1 over the three source classes. Secondary diagnostics include fault-only sensor-local/process macro-F1, ROC-AUC, PR-AUC, false alarm rate (FAR), top-1 and top-2 affected-channel shortlisting, mean reciprocal rank (MRR), and mean rank.

The experiments were implemented in Python; the public artifact records the package requirements and run metadata, including PyTorch 2.7.1+cu118 and CUDA availability on the assembly machine.

### 3.2. Data Sources and Injected Perturbations

Table 1 summarizes the evaluation protocol. The synthetic benchmark generates multichannel windows from shared oscillatory and random-walk latent components with channel-specific gains, offsets, noise, and lags. UCI HAR contributes wearable-like inertial channels. UCI Air Quality contributes environmental gas and concentration sensor channels.

Each run uses windows of length T=128, 50 training windows per fine-grained class, and 50 test windows per fine-grained class. Synthetic experiments use 240 calibration windows. UCI HAR uses subject-disjoint calibration, supervised-training, and test subject groups within the public training partition. UCI Air Quality uses a 35%/30%/35% chronological raw-series split. Interpolation is performed within each block, and missing-value fills and normalization statistics are estimated from calibration plus training blocks and applied to the test block.

Sensor-local perturbations include offset drift, scale drift, gradual trend drift, noise-floor change, saturation, dropout, and time lag. These perturbation families follow common sensor data-fault mechanisms [22]: calibration bias and gain error, slow drift, elevated measurement noise, range saturation, communication or sensing dropout, and synchronization delay. The numerical ranges are controlled stress-test settings, and the coherent multichannel process shock is a stylized process-level perturbation. For each perturbed window, the perturbation starts at a random point between 25% and 55% of the window and continues to the end. Exact parameter ranges, seeds, and command-line settings are included in the supplementary reproducibility artifact.

### 3.3. HRA Feature Construction

HRA first fits cross-channel consistency models from calibration windows. For each target channel *c*, a ridge predictor estimates $X_{:,c}$ from the remaining channels:

$${\hat{x}}_{t,c}=f_{c}\left(X_{t,-c}\right),\;r_{t,c}=x_{t,c}-{\hat{x}}_{t,c}.$$

The implementation uses α=1.0 for the ridge models and keeps this value fixed across datasets. The residual matrix $R=[r_{t,c}]$ is then transformed into the feature blocks below.

**HRA-Core diagnostic signature.** HRA-Core is the window-level residual diagnostic signature used for source-level classification. It is fitted from calibration windows by training the leave-one-channel ridge consistency models above, after per-window channel normalization. At inference time, the fitted models are applied to a candidate window to obtain residuals for all channels. HRA-Core then converts the observed window and residual matrix into a fixed-length diagnostic vector with four groups of statistics. First, it records per-channel residual magnitude and trend statistics, including residual mean-square error, mean absolute error, maximum absolute residual, and residual slope. Second, it records direct channel-behavior cues from the observed window, including zero-valued sample ratios, clipping ratios estimated from within-window quantiles, and a normalized lag cue computed by cross-correlating each channel with the mean of the remaining channels over lags from −8 to 8. Third, it records global consistency summaries, including coarse MSE-based residual-energy concentration, aggregate residual magnitude, first-difference magnitude, and upper-triangular channel-correlation statistics. Fourth, it computes a change signature by scanning 17 candidate split points between 0.2T and 0.8T and selecting the split with the largest combined change in channel means, channel standard deviations, slopes, and residual energy. For that selected split, HRA-Core stores the largest sorted absolute changes in mean, log-standard-deviation ratio, slope, residual-energy ratio, zero ratio, clipping ratio, and inter-channel correlation, together with thresholded affected-channel counts and split-level summary values. Thus, HRA-Core is a source-level diagnostic signature: it summarizes how a whole window departs from calibration-fitted cross-channel consistency, but it is not the per-channel ranking block used for localization.

**Channel attribution.** HRA computes a per-channel attribution score $a_{c}$ from residual energy and change signatures. The scan considers 17 candidate split points between 0.2T and 0.8T and is not given the injected start index. The score combines residual energy, mean shift, scale shift, slope change, noise-floor change, dropout evidence, and clipping evidence. Affected-channel localization ranks channels by $a_{c}$.

**Residual concentration.** Let $p_{c}=a_{c}/\sum_{j}a_{j}$. HRA uses the top-1 attribution mass $\max_{c}p_{c}$, the sum of the two largest attribution masses, normalized entropy $-\sum_{c}p_{c}\log\left(p_{c}\right)/\log C$, the Gini coefficient of the attribution distribution, and residual spread. These quantities summarize how concentrated the residual evidence is across channels and are passed to the diagnostic probe as explicit residual-structure features.

**Residual coherence.** HRA computes off-diagonal residual-correlation summaries, including mean and maximum absolute residual correlation. Process faults may produce coherent multichannel residual structure, but the pattern depends on dataset coupling and perturbation strength.

The feature-block ablations in Section 4.3 keep these components separate: HRA-Core is the calibration-fitted window-level diagnostic signature; attribution scores are the per-channel ranking scores; residual summaries collect per-channel residual magnitudes, maxima, slopes, and aggregate residual-change terms; and residual concentration/coherence indices are explicit summaries derived from the attribution distribution and residual-correlation matrix. The HRA + spectral configuration augments the HRA feature representation with the matched spectral feature block for source-level diagnosis. At the source-diagnosis module level, HRA is lightweight in a fitted-model footprint: the fitted HRA consistency model contains 36 ridge coefficients for the six-channel UCI HAR setting and 64 for the eight-channel synthetic and Air Quality settings. The HRA + spectral configuration adds a non-neural spectral feature block, giving 72 and 128 recorded source-module entries, respectively.

Supplementary design analyses vary the ridge parameter over α∈{0.01,0.1,1,10,100} and vary the change-signature scan across candidate counts and scan ranges. The fixed α=1.0 setting is used as a common protocol value rather than a dataset-specific tuned parameter, and the 17-point scan over [0.2T,0.8T] is retained as a stable protocol-performance compromise in the tested grid.

### 3.4. Source-Level Probe

HRA uses a two-stage logistic diagnostic probe. Stage 1 separates normal from faulty windows. Stage 2 is trained on faulty training windows and separates sensor-local from process faults. The Stage-1 threshold is selected on training windows, and standardization, feature selection, and model selection follow the predefined train/test split. Flat three-class probes are reported as a design sensitivity check that separates the value of the hierarchical workflow from the value of the feature representation.

### 3.5. Supervised Affected-Channel Shortlisting

The HRA attribution score $a_{c}$ provides an unsupervised channel ranking. The supervised localization experiment uses a dedicated supervised localizer, HRA-SL, for affected-channel candidate ranking. For each sensor-local training window, HRA-SL forms one candidate example per channel. The candidate with channel index $c^{★}$ is labeled positive and all other channel candidates are labeled negative.

The HRA-SL channel-candidate feature vector contains channel-level statistics from the observed window, HRA residual-attribution evidence, and calibration-fitted TCN-AE and USAD-like [1] reconstruction energies. Reconstruction models use calibration-split validation for early stopping and hyperparameter selection. The main HRA-SL localizer uses a fixed gradient-boosted tree classifier for all datasets, separating source-level diagnosis from affected-channel shortlisting.

### 3.6. Baselines

The baseline set covers statistical, spectral, reconstruction-feature, and supervised shortlisting alternatives as follows:

- Statistical features with the same hierarchical logistic probe;
- Spectral time-frequency features with the same hierarchical logistic probe;
- Statistical + spectral feature fusion with the same hierarchical logistic probe;
- Tuned TCN-AE and tuned USAD-like [1] reconstruction-feature baselines;
- A GRU-AE reconstruction-feature baseline;
- Supervised channel-localizer comparators, including AE-only reconstruction-energy localizers.

The reconstruction baselines use calibration-split fitting, reconstruction-error features, and the same downstream hierarchical probe. The supervised channel-localizer baselines use the same target-channel protocol as HRA-SL. Supplementary Table S5 documents layer counts, hidden dimensions, kernel or recurrent settings, optimizer settings, learning-rate grid, epoch budgets, early stopping, seeds, selection criterion, and retained parameter counts for TCN-AE, USAD-like AE, GRU-AE, and adapted TranAD. Supplementary Table S4 reports the adapted official TranAD reconstruction-evidence evaluation under the same split and downstream-probe constraints.

## 4. Results

### 4.1. Source-Level Baseline Comparison

**Figure 2:**
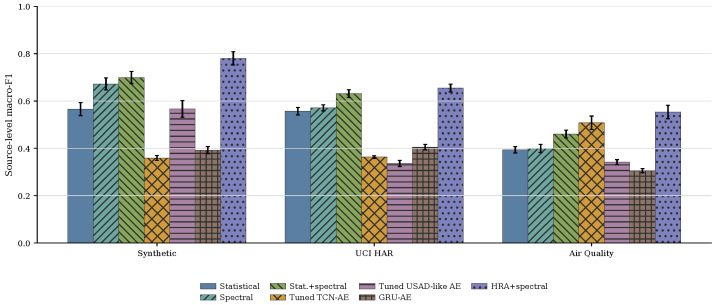
Source-level macro-F1 for evaluated baselines. Error bars show 95% confidence intervals over the matched seed/severity matrix. Localization comparisons are reported separately in Table 3.

Table 2 reports the source-level baseline comparison. HRA + spectral provides the main HRA source-level configuration and improves over the evaluated baselines across the three datasets. It reaches 0.780±0.028, 0.655±0.017, and 0.554±0.029 source-level macro-F1 on synthetic windows, UCI HAR, and Air Quality, respectively. Relative to tuned TCN-AE, HRA + spectral improves source-level macro-F1 by 0.421, 0.292, and 0.046 on synthetic, UCI HAR, and Air Quality, respectively, using paired bootstrap differences over matched seed/severity cells. Figure 2 visualizes the same source-level macro-F1 comparison across the evaluated baselines.

### 4.2. Supervised Channel Shortlisting

Table 3 reports the HRA-SL localization experiment together with matched supervised and unsupervised ranking comparators. The fixed HGB HRA-SL localizer, using HRA residual and reconstruction-energy channel-candidate features, reaches top-1 affected-channel localization of 0.916 on synthetic data, 0.714 on UCI HAR, and 0.715 on Air Quality. Under matched target-channel supervision, AE-only HGB is practically tied with HRA-SL on synthetic data and UCI HAR, and HRA-SL is higher on Air Quality. Against the strongest unsupervised tuned reconstruction ranking baseline, HRA-SL improves top-1 localization by +0.127 on synthetic data (95% CI [0.117, 0.137]), +0.357 on UCI HAR (95% CI [0.344, 0.370]), and +0.048 on Air Quality (95% CI [0.033, 0.063]). Table 4 reports top-2 values and confidence intervals for the matched supervised comparison.

The matched supervised comparison in Table 4 shows that AE-only HGB is a strong task-specific comparator: it uses training-split target-channel labels, per-channel reconstruction-energy cues from TCN-AE and USAD-like models, and the same HGB candidate classifier. On synthetic and UCI HAR data, HRA-SL and AE-only HGB are practical ties at top-1 (0.916 versus 0.918, and 0.714 versus 0.716). Their top-2 and MRR values are also close on both datasets. On Air Quality, where shared environmental drift produces a more heterogeneous localization setting, HRA-SL is higher than AE-only HGB on top-1 (0.715 versus 0.652), top-2 (0.849 versus 0.820), and MRR (0.822 versus 0.783). These results make the complementarity claim dataset- and classifier-dependent: HRA residual cues and AE reconstruction-energy cues are most clearly complementary for Air Quality, while the synthetic and UCI HAR matched-supervision comparisons are effectively saturated.

### 4.3. Feature-Block Ablations

Table 5 decomposes HRA into its central source-level component and individual residual-structure feature blocks. The calibration-fitted diagnostic signature is the HRA-Core source-level component, summarizing deviations from normal cross-channel consistency using calibration-fitted statistics. This representation carries much of the source-level information, especially on synthetic and UCI HAR data. Residual summaries, attribution scores, and explicit concentration/coherence indices provide interpretable residual-structure evidence, while HRA + spectral gives the main fused source-level configuration.

Relative to HRA-Core, HRA + spectral improves source-level macro-F1 by about +0.062 on synthetic data and +0.034 on UCI HAR while remaining competitive on Air Quality.

**Figure 3:**
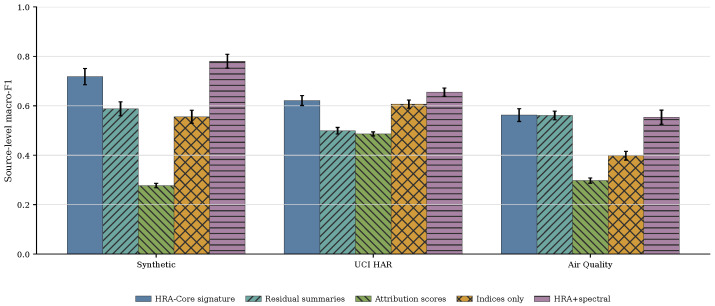
Feature-block ablation for source-level macro-F1. The HRA-Core diagnostic signature carries much of the source-level signal, while expanded residual features and spectral fusion provide complementary dataset-dependent changes.

As a design sensitivity check, flat three-class probes are reported in Supplementary Materials. HRA + spectral with the hierarchical probe is essentially tied with the flat probe on synthetic data, lower on UCI HAR, and slightly higher on Air Quality. This confirms that the source-level performance is mainly carried by the HRA-Core/HRA + spectral representation, while the hierarchy is retained to organize the alarm-source diagnostic workflow. Figure 3 summarizes the feature-block ablation for source-level macro-F1.

### 4.4. Fault-Type Analysis

**Figure 4:**
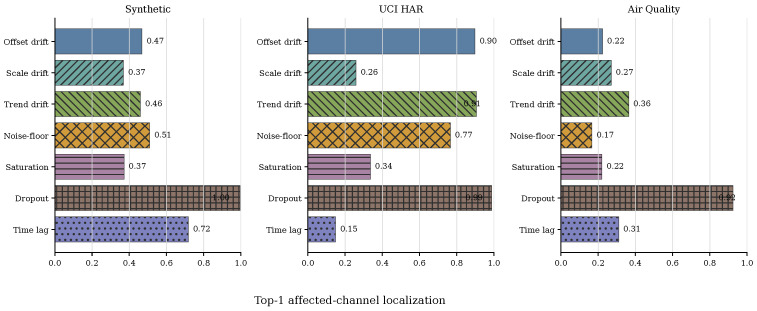
Fine-grained sensor-local perturbation breakdown using HRA attribution scores. Bars show top-1 affected-channel localization for each injected sensor-local fault type.

The fine-grained perturbation analysis reported from the supplementary fault-type localization table shows which injected faults drive the channel-ranking signal. Dropout is localized well under the HRA attribution score in all three datasets: top-1 localization is 0.996, 0.988, and 0.924 on synthetic, UCI HAR, and Air Quality. Offset and trend drift are also strong on UCI HAR, with top-1 localization of 0.898 and 0.906, respectively. Saturation and scale drift are harder on UCI HAR, while noise-floor, offset, saturation, and scale perturbations are harder on Air Quality. These patterns also explain why the unsupervised HRA attribution score is weaker than reconstruction-energy ranking on synthetic and Air Quality for some local faults. Single-channel synthetic perturbations often create a direct reconstruction-energy peak at the affected channel, while the HRA score intentionally balances residual energy with change, concentration, and coherence cues for source diagnosis. In Air Quality, chronological distribution shift in PT08.S4(NO2), NO2(GT), and NOx(GT), together with shared environmental variation where the first two principal components explain about 0.89 of test-set variance, disperses residual evidence across correlated channels. HRA attribution is therefore most useful as interpretable channel evidence and as an input to HRA-SL, not as a universally strongest unsupervised localizer. Figure 4 visualizes the fine-grained top-1 localization breakdown by injected sensor-local fault type.

### 4.5. Design-Choice Analyses

Supplementary Tables S1–S6 summarize the design-choice, baseline, and diagnostic analyses. In the ridge sweep over α∈{0.01,0.1,1,10,100}, HRA + spectral source-level macro-F1 changes only narrowly on synthetic data and UCI HAR, with rounded ranges of 0.780–0.781 and 0.654–0.656, respectively; Air Quality ranges from 0.549 to 0.575, with α=1 giving 0.554. HRA-Core is stable over the same grid, with macro-F1 spreads of 0.004, 0.001, and 0.009 on synthetic, UCI HAR, and Air Quality.

The split-scan analysis compares candidate counts and scan ranges without using the injected start index. In the retained [0.2T,0.8T] scan, the 17-point HRA + spectral setting remains within the main-result range on all three datasets and within 0.003 of the best 5/17/65-point setting, while saving about 4.1–4.3 s per seed/severity cell compared with 65 points. The retained scan is therefore a protocol-design compromise rather than a dataset-tuned optimum.

An adapted official TranAD reconstruction-evidence evaluation is reported in Supplementary Table S4: its source-level macro-F1 is 0.354, 0.383, and 0.304 on synthetic, UCI HAR, and Air Quality under the same downstream diagnostic probe.

## 5. Discussion

The experiments support a three-step diagnostic workflow: fault detection, source-level diagnosis, and affected-channel shortlisting. This workflow is more actionable than binary anomaly detection because it links sensor-local and process-level events to different operational responses.

The ablation results show that HRA-Core carries most of the source-level signal, while residual summaries, concentration/coherence indices, and attribution scores add targeted information for interpretation and channel ranking. Spectral fusion helps most clearly on synthetic and UCI HAR data, where time-frequency structure is discriminative. Air Quality shows a different response profile, with chronological distribution shift and shared environmental background variation making spectral fusion less decisive.

The reconstruction baselines sharpen this interpretation. Tuned TCN-AE, tuned USAD-like AE, and GRU-AE baselines trail HRA + spectral on source-level macro-F1, but reconstruction-energy cues remain useful for localizing injected single-channel perturbations. Their advantage in some unsupervised ranking cells is expected: reconstruction energy is a direct per-channel error cue, whereas HRA attribution distributes evidence across residual magnitude, local change, concentration, and coherence so that the same representation can support source-level diagnosis. HRA-SL therefore combines HRA residual features with AE reconstruction-energy cues in a supervised candidate ranking model. For affected-channel shortlisting, HRA-SL outperformed the strongest unsupervised tuned reconstruction ranking baseline on all three datasets, matched the strongest supervised AE-only localizer in practical terms on synthetic and UCI HAR, and was higher on Air Quality.

The controlled-injection setting is also a practical evaluation device. Public sensor datasets rarely contain both source labels and affected-channel labels, while injected perturbations make those labels available under a fixed split and severity matrix. This design turns public base signals into a repeatable stress test for three operational questions: whether a window is abnormal, whether the source is sensor-local or process-level, and which channels should be inspected first. Field studies with naturally occurring failures, repair logs, and changing operating regimes are the next validation stage; the present benchmark provides a clean pre-deployment basis for selecting and evaluating candidate diagnostic workflows.

## 6. Conclusions

This paper presented HRA-SL as a residual-attribution and supervised shortlisting framework for multivariate sensor diagnostics. HRA provides the source-level residual-diagnosis component, HRA + spectral is the main fused source-diagnosis configuration, and HRA-SL extends this pipeline with supervised affected-channel shortlisting. In a controlled injected-perturbation evaluation on synthetic, UCI HAR, and UCI Air Quality base signals, HRA + spectral improved source-level macro-F1 over evaluated statistical, spectral, statistical + spectral, tuned reconstruction-feature, GRU-AE, and adapted TranAD reconstruction-evidence baselines under a matched diagnostic-probe protocol. The ablations identify HRA-Core as the dominant source-level component and residual-attribution features as channel-ranking and diagnostic-interpretation evidence. For affected-channel shortlisting, HRA-SL outperformed the strongest unsupervised tuned reconstruction ranking baseline on all three datasets, matched the strongest supervised AE-only localizer in practical terms on synthetic and UCI HAR, and was higher on Air Quality.

At the source-diagnosis module level, HRA remains lightweight in a fitted-model footprint, using 36–64 ridge coefficients; with the non-neural spectral block, HRA + spectral has 72–128 recorded source-module entries across the evaluated channel counts.

The conclusions are bounded by the controlled perturbation protocol: source labels and target-channel labels are generated by injection, HRA-SL uses training-split target-channel labels for supervised shortlisting, and held-out labels quantify localization. Public datasets provide reproducible base dynamics; field studies with naturally occurring hardware failures, repair actions, and long-term regime changes are an important follow-up setting. The main deployment condition is learnable cross-channel consistency, so weakly coupled, delayed, or strongly nonstationary systems may benefit from physical consistency models or additional features. Within this scope, HRA-SL offers a practical and reproducible workflow for early-stage sensor-system diagnostic evaluation.

## Figures and Tables

**Table 1:** Evaluation protocol. Public-data experiments split the base signals before perturbation injection, with preprocessing and normalization fitted from the calibration and training portions.

Dataset	Channels	Main Matrix	Severities	Split Protocol
Synthetic	8	5 seeds × 5 severities	0.75–1.75	independent generation
UCI HAR	6	5 seeds × 5 severities	0.75–1.75	subject-disjoint split
UCI Air Quality	8	5 seeds × 5 severities	0.75–1.75	chronological split

**Table 2:** Source-level baseline summary. Macro-F1 is source-level macro-F1 over normal, sensor-local fault, and process fault classes. Fault-source F1 is computed over the two faulty source classes. F1 values are means ±95% CI half-width over the predefined 25 seed/severity runs; FAR is reported as the mean false-alarm rate. Localization results are reported separately in Section 4.2 and Table 3.

Dataset	Method	Macro-F1	Fault-Source F1	FAR
Synthetic	Statistical	0.566 ± 0.027	0.767 ± 0.029	0.549
Synthetic	Spectral	0.672 ± 0.025	0.763 ± 0.024	0.490
Synthetic	Stat. + spectral	0.699 ± 0.025	0.800 ± 0.024	0.466
Synthetic	Tuned TCN-AE	0.359 ± 0.010	0.496 ± 0.013	0.635
Synthetic	Tuned USAD-like AE	0.567 ± 0.035	0.718 ± 0.035	0.354
Synthetic	GRU-AE	0.392 ± 0.015	0.551 ± 0.017	0.721
Synthetic	HRA + spectral	0.780 ± 0.028	0.903 ± 0.023	0.458
UCI HAR	Statistical	0.557 ± 0.016	0.828 ± 0.019	0.756
UCI HAR	Spectral	0.571 ± 0.013	0.798 ± 0.013	0.761
UCI HAR	Stat. + spectral	0.631 ± 0.016	0.875 ± 0.014	0.766
UCI HAR	Tuned TCN-AE	0.363 ± 0.005	0.558 ± 0.006	0.867
UCI HAR	Tuned USAD-like AE	0.336 ± 0.013	0.515 ± 0.015	0.770
UCI HAR	GRU-AE	0.405 ± 0.011	0.602 ± 0.018	0.802
UCI HAR	HRA + spectral	0.655 ± 0.017	0.907 ± 0.013	0.770
Air Quality	Statistical	0.394 ± 0.014	0.576 ± 0.016	0.472
Air Quality	Spectral	0.400 ± 0.017	0.588 ± 0.020	0.723
Air Quality	Stat. + spectral	0.461 ± 0.016	0.647 ± 0.024	0.711
Air Quality	Tuned TCN-AE	0.508 ± 0.028	0.629 ± 0.023	0.454
Air Quality	Tuned USAD-like AE	0.342 ± 0.011	0.522 ± 0.012	0.801
Air Quality	GRU-AE	0.305 ± 0.009	0.477 ± 0.012	0.786
Air Quality	HRA + spectral	0.554 ± 0.029	0.699 ± 0.025	0.611

**Table 3:** Affected-channel shortlisting comparison. HRA-SL and AE-only HGB use target-channel labels in the supervised-training split with the fixed HGB candidate classifier. HRA attribution ranking provides the unsupervised residual-attribution comparator, while tuned TCN-AE and tuned USAD-like AE provide unsupervised reconstruction-ranking comparators.

Dataset	Ranking Method	Supervision	Top-1 Loc.	MRR
Synthetic	HRA-SL (HGB)	supervised	0.916	0.950
	AE-only HGB	supervised	0.918	0.949
	HRA attribution ranking	unsupervised HRA ranking	0.556	0.650
	Tuned TCN-AE	unsupervised reconstruction	0.462	0.622
	Tuned USAD-like AE	unsupervised reconstruction	0.789	0.866
UCI HAR	HRA-SL (HGB)	supervised	0.714	0.812
	AE-only HGB	supervised	0.716	0.812
	HRA attribution ranking	unsupervised HRA ranking	0.614	0.737
	Tuned TCN-AE	unsupervised reconstruction	0.357	0.556
	Tuned USAD-like AE	unsupervised reconstruction	0.253	0.454
Air Quality	HRA-SL (HGB)	supervised	0.715	0.822
	AE-only HGB	supervised	0.652	0.783
	HRA attribution ranking	unsupervised HRA ranking	0.354	0.534
	Tuned TCN-AE	unsupervised reconstruction	0.666	0.785
	Tuned USAD-like AE	unsupervised reconstruction	0.296	0.473

**Table 4:** Matched supervised localizer comparison. Values are affected-channel localization means ±95% CI half-width over the predefined 25 seed/severity runs. HRA-SL (HGB) uses the HRA + AE channel-candidate feature block with the fixed HGB classifier, and AE-only HGB uses the same target-channel supervision and classifier with reconstruction-energy channel cues.

Dataset	Supervised Localizer	Top-1 Loc.	Top-2 Loc.	MRR
Synthetic	HRA-SL (HGB)	0.916 ± 0.023	0.964 ± 0.014	0.950 ± 0.014
	AE-only HGB	0.918 ± 0.024	0.957 ± 0.015	0.949 ± 0.016
UCI HAR	HRA-SL (HGB)	0.714 ± 0.011	0.818 ± 0.008	0.812 ± 0.007
	AE-only HGB	0.716 ± 0.009	0.814 ± 0.008	0.812 ± 0.006
Air Quality	HRA-SL (HGB)	0.715 ± 0.030	0.849 ± 0.024	0.822 ± 0.021
	AE-only HGB	0.652 ± 0.026	0.820 ± 0.026	0.783 ± 0.019

**Table 5:** Source-level HRA feature-block ablation. The table separates the calibration-fitted HRA-Core diagnostic signature from individual residual summaries, attribution scores, explicit residual concentration/coherence indices, and the HRA + spectral source-level configuration. Macro-F1 and fault-source F1 are means ± 95% CI half-width over 25 seed/severity runs; FAR is reported as the mean false-alarm rate. Channel ranking and supervised affected-channel shortlisting are evaluated separately in Table 3.

Dataset	Method	Macro-F1	Fault-Source F1	FAR
Synthetic	HRA-Core signature	0.718 ± 0.033	0.875 ± 0.030	0.506
Synthetic	Residual summaries	0.588 ± 0.028	0.754 ± 0.028	0.558
Synthetic	Attribution scores	0.277 ± 0.009	0.495 ± 0.014	0.602
Synthetic	Indices only	0.556 ± 0.026	0.696 ± 0.028	0.557
Synthetic	HRA+spectral	0.780 ± 0.028	0.903 ± 0.023	0.458
UCI HAR	HRA-Core signature	0.621 ± 0.020	0.880 ± 0.018	0.711
UCI HAR	Residual summaries	0.499 ± 0.013	0.740 ± 0.017	0.706
UCI HAR	Attribution scores	0.486 ± 0.008	0.679 ± 0.008	0.714
UCI HAR	Indices only	0.607 ± 0.017	0.849 ± 0.018	0.684
UCI HAR	HRA+spectral	0.655 ± 0.017	0.907 ± 0.013	0.770
Air Quality	HRA-Core signature	0.562 ± 0.026	0.707 ± 0.025	0.476
Air Quality	Residual summaries	0.561 ± 0.018	0.705 ± 0.016	0.480
Air Quality	Attribution scores	0.297 ± 0.011	0.484 ± 0.016	0.686
Air Quality	Indices only	0.398 ± 0.018	0.583 ± 0.010	0.899
Air Quality	HRA+spectral	0.554 ± 0.029	0.699 ± 0.025	0.611

## Data Availability

The UCI HAR and UCI Air Quality datasets are publicly available from the UCI Machine Learning Repository. Synthetic data and injected perturbations are generated by the supplementary scripts. Processed result tables and figure-generation inputs are included in the supplementary artifact. The public artifact repository is available at https://github.com/GeniusWanghaha/hra_sl_code_source (accessed on 1 August 2026).

## Supplementary Materials

The following supporting information can be downloaded at https://www.mdpi.com/article/10.3390/s26154986/s1: Table S1: Ridge-alpha sweep; Table S2: Split-scan analysis; Table S3: Hierarchy/flat design sensitivity; Table S4: Adapted TranAD comparison; Table S5: Deep-baseline hyperparameters; Table S6: Air Quality diagnostic summaries. The supplementary artifact contains experiment code, per-run CSV/JSON files, generated tables, figure inputs, perturbation settings, random seeds, and reproduction commands. The public artifact repository is available at https://github.com/GeniusWanghaha/hra_sl_code_source (accessed on 1 August 2026).

## Abbreviations

AE	Autoencoder
FAR	False alarm rate
HRA	Hierarchical Residual Attribution
HRA-SL	Hierarchical Residual Attribution with supervised channel shortlisting
MRR	Mean reciprocal rank
RCI	Residual concentration index
RCS	Residual coherence score
TCN	Temporal convolutional network
USAD	UnSupervised Anomaly Detection

## References

[1] Audibert J., Michiardi P., Guyard F., Marti S., Zuluaga M.A., Gupta R., Liu Y., Tang J., Prakash B.A. (2020). USAD: UnSupervised Anomaly Detection on Multivariate Time Series. Proceedings of the KDD ’20: The 26th ACM SIGKDD Conference on Knowledge Discovery and Data Mining, Virtual Event, CA, USA, 23–27 August 2020.

[2] Su Y., Zhao Y., Niu C., Liu R., Sun W., Pei D., Teredesai A., Kumar V., Li Y., Rosales R., Terzi E., Karypis G. (2019). Robust Anomaly Detection for Multivariate Time Series through Stochastic Recurrent Neural Network. Proceedings of the 25th ACM SIGKDD International Conference on Knowledge Discovery & Data Mining, KDD 2019, Anchorage, AK, USA, 4–8 August 2019.

[3] Deng A., Hooi B. (2021). Graph Neural Network-Based Anomaly Detection in Multivariate Time Series. Proceedings of the Thirty-Fifth AAAI Conference on Artificial Intelligence, AAAI 2021, Thirty-Third Conference on Innovative Applications of Artificial Intelligence, IAAI 2021, The Eleventh Symposium on Educational Advances in Artificial Intelligence, EAAI 2021, Virtual Event, 2–9 February 2021.

[4] Tuli S., Casale G., Jennings N.R. (2022). TranAD: Deep Transformer Networks for Anomaly Detection in Multivariate Time Series Data. Proc. VLDB Endow..

[5] Xu J., Wu H., Wang J., Long M. (2022). Anomaly Transformer: Time Series Anomaly Detection with Association Discrepancy. Proceedings of the Tenth International Conference on Learning Representations, ICLR 2022, Virtual Event, 25–29 April 2022.

[6] Li Z., Zhao Y., Han J., Su Y., Jiao R., Wen X., Pei D., Zhu F., Ooi B.C., Miao C. (2021). Multivariate Time Series Anomaly Detection and Interpretation using Hierarchical Inter-Metric and Temporal Embedding. Proceedings of the KDD ’21: The 27th ACM SIGKDD Conference on Knowledge Discovery and Data Mining, Virtual Event, Singapore, 14–18 August 2021.

[7] Ren H., Xu B., Wang Y., Yi C., Huang C., Kou X., Xing T., Yang M., Tong J., Zhang Q., Teredesai A., Kumar V., Li Y., Rosales R., Terzi E., Karypis G. (2019). Time-Series Anomaly Detection Service at Microsoft. Proceedings of the 25th ACM SIGKDD International Conference on Knowledge Discovery & Data Mining, KDD 2019, Anchorage, AK, USA, 4–8 August 2019.

[8] Geiger A., Liu D., Alnegheimish S., Cuesta-Infante A., Veeramachaneni K., Wu X., Jermaine C., Xiong L., Hu X., Kotevska O., Lu S., Xu W., Aluru S., Zhai C., Al-Masri E. (2020). TadGAN: Time Series Anomaly Detection Using Generative Adversarial Networks. Proceedings of the 2020 IEEE International Conference on Big Data (IEEE BigData 2020), Atlanta, GA, USA, 10–13 December 2020.

[9] Li D., Chen D., Jin B., Shi L., Goh J., Ng S., Tetko I.V., Kurková V., Karpov P., Theis F.J. (2019). MAD-GAN: Multivariate Anomaly Detection for Time Series Data with Generative Adversarial Networks. Proceedings of the Artificial Neural Networks and Machine Learning—ICANN 2019: Text and Time Series—28th International Conference on Artificial Neural Networks, Munich, Germany, 17–19 September 2019.

[10] Zhang Z., Li W., Ding W., Zhang L., Lu Q., Hu P., Gui T., Lu S. (2023). STAD-GAN: Unsupervised Anomaly Detection on Multivariate Time Series with Self-training Generative Adversarial Networks. ACM Trans. Knowl. Discov. Data.

[11] Yang Y., Zhang C., Zhou T., Wen Q., Sun L., Singh A.K., Sun Y., Akoglu L., Gunopulos D., Yan X., Kumar R., Ozcan F., Ye J. (2023). DCdetector: Dual Attention Contrastive Representation Learning for Time Series Anomaly Detection. Proceedings of the 29th ACM SIGKDD Conference on Knowledge Discovery and Data Mining, KDD 2023, Long Beach, CA, USA, 6–10 August 2023.

[12] Xu H., Pang G., Wang Y., Wang Y. (2023). Deep Isolation Forest for Anomaly Detection. IEEE Trans. Knowl. Data Eng..

[13] Chen Z., Chen D., Zhang X., Yuan Z., Cheng X. (2022). Learning Graph Structures With Transformer for Multivariate Time-Series Anomaly Detection in IoT. IEEE Internet Things J..

[14] Han X., Absar S., Zhang L., Yuan S. (2025). Root Cause Analysis of Anomalies in Multivariate Time Series through Granger Causal Discovery. Proceedings of the Thirteenth International Conference on Learning Representations, ICLR 2025, Singapore, 24–28 April 2025.

[15] Blázquez-García A., Conde A., Mori U., Lozano J.A. (2022). A Review on Outlier/Anomaly Detection in Time Series Data. ACM Comput. Surv..

[16] Darban Z.Z., Webb G.I., Pan S., Aggarwal C., Salehi M. (2025). Deep Learning for Time Series Anomaly Detection: A Survey. ACM Comput. Surv..

[17] Schmidl S., Wenig P., Papenbrock T. (2022). Anomaly Detection in Time Series: A Comprehensive Evaluation. Proc. VLDB Endow..

[18] Lai K., Zha D., Xu J., Zhao Y., Wang G., Hu X.B., Vanschoren J., Yeung S. (2021). Revisiting Time Series Outlier Detection: Definitions and Benchmarks. Proceedings of the Neural Information Processing Systems Track on Datasets and Benchmarks 1, NeurIPS Datasets and Benchmarks 2021, Virtual, 6–14 December 2021.

[19] Cook A.A., Misirli G., Fan Z. (2020). Anomaly Detection for IoT Time-Series Data: A Survey. IEEE Internet Things J..

[20] Isermann R. (2005). Model-based fault-detection and diagnosis—Status and applications. Annu. Rev. Control..

[21] Qin S.J. (2012). Survey on data-driven industrial process monitoring and diagnosis. Annu. Rev. Control..

[22] Ni K., Ramanathan N., Chehade M.N.H., Balzano L., Nair S., Zahedi S., Kohler E., Pottie G.J., Hansen M.H., Srivastava M.B. (2009). Sensor network data fault types. ACM Trans. Sens. Netw..

[23] LaRosa N., Farber J., Venkitasubramaniam P., Blum R.S., Rashdan A.A. (2022). Separating Sensor Anomalies From Process Anomalies in Data-Driven Anomaly Detection. IEEE Signal Process. Lett..

[24] Yan P., Abdulkadir A., Luley P., Rosenthal M., Schatte G.A., Grewe B.F., Stadelmann T. (2024). A Comprehensive Survey of Deep Transfer Learning for Anomaly Detection in Industrial Time Series: Methods, Applications, and Directions. IEEE Access.

[25] Gao T., Yang J., Wang W., Fan X. (2024). A domain feature decoupling network for rotating machinery fault diagnosis under unseen operating conditions. Reliab. Eng. Syst. Saf..

[26] Li Y., Yang J., Wang W., Gao T. (2026). A joint collaborative adaptation network for fault diagnosis of rolling bearing under class imbalance and variable operating conditions. Adv. Eng. Inform..

[27] Liu S., Ji Z., Zhang Z., Wang Y. (2024). An Improved Deep Transfer Learning Method for Rotating Machinery Fault Diagnosis Based on Time Frequency Diagram and Pretraining Model. IEEE Trans. Instrum. Meas..

[28] Zhou Y., Liang M., Yue X. (2024). Deep residual learning for acoustic emission source localization in a steel-concrete composite slab. Constr. Build. Mater..

[29] Sun J., Yu Z., Xu C., Du F. (2024). Experimental and Numerical Investigation of Acoustic Emission Source Localization Using an Enhanced Guided Wave Phased Array Method. Sensors.

[30] Anguita D., Ghio A., Oneto L., Parra X., Reyes-Ortiz J.L. A Public Domain Dataset for Human Activity Recognition using Smartphones. Proceedings of the 21st European Symposium on Artificial Neural Networks, ESANN 2013.

[31] Mathur A.P., Tippenhauer N.O. (2016). SWaT: A water treatment testbed for research and training on ICS security. Proceedings of the 2016 International Workshop on Cyber-Physical Systems for Smart Water Networks, CySWater@CPSWeek 2016, Vienna, Austria, 11 April 2016.

[32] Yue Z., Wang Y., Duan J., Yang T., Huang C., Tong Y., Xu B. (2022). TS2Vec: Towards Universal Representation of Time Series. Proceedings of the Thirty-Sixth AAAI Conference on Artificial Intelligence, AAAI 2022, Thirty-Fourth Conference on Innovative Applications of Artificial Intelligence, IAAI 2022, The Twelveth Symposium on Educational Advances in Artificial Intelligence, EAAI 2022 Virtual Event, 22 February–1 March 2022.

[33] Woo G., Liu C., Sahoo D., Kumar A., Hoi S.C.H. (2022). CoST: Contrastive Learning of Disentangled Seasonal-Trend Representations for Time Series Forecasting. Proceedings of the Tenth International Conference on Learning Representations, ICLR 2022, Virtual Event, 25–29 April 2022.

[34] Zhang X., Zhao Z., Tsiligkaridis T., Zitnik M., Koyejo S., Mohamed S., Agarwal A., Belgrave D., Cho K., Oh A. (2022). Self-Supervised Contrastive Pre-Training For Time Series via Time-Frequency Consistency. Proceedings of the Advances in Neural Information Processing Systems 35: Annual Conference on Neural Information Processing Systems 2022, NeurIPS 2022, New Orleans, LA, USA, 28 November–9 December 2022.

[35] Liu J., Chen S., Wooldridge M.J., Dy J.G., Natarajan S. (2024). TimesURL: Self-Supervised Contrastive Learning for Universal Time Series Representation Learning. Proceedings of the Thirty-Eighth AAAI Conference on Artificial Intelligence, AAAI 2024, Thirty-Sixth Conference on Innovative Applications of Artificial Intelligence, IAAI 2024, Fourteenth Symposium on Educational Advances in Artificial Intelligence, EAAI 2024, Vancouver, BC, Canada, 20–27 February 2024.

[36] Bonifati A., Buono F.D., Guerra F., Tiano D. (2022). Time2Feat: Learning Interpretable Representations for Multivariate Time Series Clustering. Proc. VLDB Endow..

[37] Le X., Luo L., Aickelin U., Tran M., Baeza-Yates R., Bonchi F. (2024). ShapeFormer: Shapelet Transformer for Multivariate Time Series Classification. Proceedings of the 30th ACM SIGKDD Conference on Knowledge Discovery and Data Mining, KDD 2024, Barcelona, Spain, 25–29 August 2024.

[38] Zhou H., Zhang S., Peng J., Zhang S., Li J., Xiong H., Zhang W. (2021). Informer: Beyond Efficient Transformer for Long Sequence Time-Series Forecasting. Proceedings of the Thirty-Fifth AAAI Conference on Artificial Intelligence, AAAI 2021, Thirty-Third Conference on Innovative Applications of Artificial Intelligence, IAAI 2021, The Eleventh Symposium on Educational Advances in Artificial Intelligence, EAAI 2021, Virtual Event, 2–9 February 2021.

[39] Nie Y., Nguyen N.H., Sinthong P., Kalagnanam J. (2023). A Time Series is Worth 64 Words: Long-term Forecasting with Transformers. Proceedings of the Eleventh International Conference on Learning Representations, ICLR 2023, Kigali, Rwanda, 1–5 May 2023.

[40] Wu H., Hu T., Liu Y., Zhou H., Wang J., Long M. (2023). TimesNet: Temporal 2D-Variation Modeling for General Time Series Analysis. Proceedings of the Eleventh International Conference on Learning Representations, ICLR 2023, Kigali, Rwanda, 1–5 May 2023.

[41] Ansari A.F., Stella L., Türkmen A.C., Zhang X., Mercado P., Shen H., Shchur O., Rangapuram S.S., Pineda-Arango S., Kapoor S. (2024). Chronos: Learning the Language of Time Series. Trans. Mach. Learn. Res..

[42] Goswami M., Szafer K., Choudhry A., Cai Y., Li S., Dubrawski A., Salakhutdinov R., Kolter Z., Heller K.A., Weller A., Oliver N., Scarlett J., Berkenkamp F. (2024). MOMENT: A Family of Open Time-series Foundation Models. Proceedings of the Forty-first International Conference on Machine Learning, ICML 2024, Vienna, Austria, 21–27 July 2024.

[43] Liu Y., Zhang H., Li C., Huang X., Wang J., Long M., Salakhutdinov R., Kolter Z., Heller K.A., Weller A., Oliver N., Scarlett J., Berkenkamp F. (2024). Timer: Generative Pre-trained Transformers Are Large Time Series Models. Proceedings of the Forty-first International Conference on Machine Learning, ICML 2024, Vienna, Austria, 21–27 July 2024.

[44] Karim F., Majumdar S., Darabi H. (2019). Insights Into LSTM Fully Convolutional Networks for Time Series Classification. IEEE Access.

[45] Dempster A., Petitjean F., Webb G.I. (2020). ROCKET: Exceptionally fast and accurate time series classification using random convolutional kernels. Data Min. Knowl. Discov..

