# Supplementary material for: Hierarchical Residual Attribution with Supervised Channel Shortlisting for Sensor-Local and Process Fault Diagnosis in Multivariate Sensor Time Series
Source: Sensors (Basel). 2026 Aug 6;26(15):4986. doi: 10.3390/s26154986 (PMC13469677; doi:10.3390/s26154986)
Supplement: Supplementary file 1 [file sensors-26-04986-s001.zip › sensors-4449958-supplementary.pdf]

Supplementary Tables

Table S1. Ridge-alpha sensitivity for HRA-Core and HRA+spectral. Values are source-level macro-F1 means over 25 matched seed/severity cells.

Dataset	HRA-Core at $\alpha = 1$	HRA-Core range	HRA+spectral at $\alpha = 1$	HRA+spectral range
Synthetic	0.715	0.711–0.716	0.780	0.780–0.781
UCI HAR	0.621	0.621–0.622	0.656	0.654–0.656
Air Quality	0.563	0.554–0.563	0.554	0.549–0.575

Table S2. Retained split-scan comparison over $[0.2T, 0.8T]$. Values report source-level macro-F1 and mean total runtime in seconds per seed/severity cell.

Dataset	Method	5 points	17 points	65 points
Synthetic	HRA-Core	0.709 / 27.9 s	0.715 / 28.9 s	0.721 / 33.2 s
Synthetic	HRA+spectral	0.778 / 27.9 s	0.780 / 28.9 s	0.779 / 33.2 s
UCI HAR	HRA-Core	0.623 / 22.1 s	0.621 / 23.2 s	0.628 / 27.3 s
UCI HAR	HRA+spectral	0.657 / 22.1 s	0.656 / 23.2 s	0.659 / 27.3 s
Air Quality	HRA-Core	0.560 / 28.4 s	0.563 / 29.5 s	0.562 / 33.7 s
Air Quality	HRA+spectral	0.550 / 28.4 s	0.554 / 29.5 s	0.555 / 33.7 s

Table S3. Hierarchical versus flat probe design sensitivity for the HRA+spectral source-diagnosis configuration. Values are computed over 25 matched seed/severity cells per dataset.

Dataset	Hierarchical probe	Flat probe	Paired Δ hierarchy–flat
Synthetic	0.780	0.780	$+0.000\ [-0.005, 0.006]$
UCI HAR	0.655	0.673	$-0.018\ [-0.026, -0.010]$
Air Quality	0.554	0.546	$+0.007\ [-0.003, 0.018]$

Table S4. Adapted official TranAD reconstruction-evidence comparison. TranAD is adapted as a reconstruction-evidence generator and uses the same downstream diagnostic probe; it is not treated as a native source-diagnosis or shortlisting model.

Dataset	TranAD F1	HRA+spec. F1	Paired Δ	Unsup. Top-1	Sup. HGB Top-1
Synthetic	0.354 ± 0.012	0.769	$-0.415\ [-0.433, -0.395]$	0.155	0.756
UCI HAR	0.383 ± 0.015	0.656	$-0.273\ [-0.282, -0.263]$	0.159	0.717
Air Quality	0.304 ± 0.020	0.553	$-0.250\ [-0.279, -0.222]$	0.117	0.511

Table S5. Deep reconstruction baseline architecture and training-setup summary. The retained rows were selected from the documented grid using training-side calibration validation; all runs used the predefined seed/severity matrix.

Method	Architecture	Search grid	Selection protocol	Retained parameters
TCN-AE	4 temporal-convolution layers; kernel size 5; ReLU; retained hidden width 48	hidden width 24/48; learning rate 10^{-3} or $5{\times}10^{-4}$; epoch budgets 10/30/50/100; Adam	batch size 64; calibration-validation loss; early-stopping patience 12; best epoch recorded	27,032 for 8-channel datasets; 26,090 in the UCI HAR full-pipeline count
USAD-like AE	4-layer encoder/decoder; ReLU; retained hidden 256 and latent 64	hidden 128/256; latent 32/64; learning rate 10^{-3} or $5{\times}10^{-4}$; epoch budgets 10/30/50/100; Adam	batch size 64; calibration-validation loss; early-stopping patience 12; best epoch recorded	558,656 for 8-channel datasets; 427,348 in the UCI HAR full-pipeline count
GRU-AE	3 recurrent layers; GRU gates; retained hidden dimension 64	hidden 32/64; learning rate 10^{-3} or $5{\times}10^{-4}$; epoch budgets 10/30/50/100; Adam	batch size 64; calibration-validation loss; early-stopping patience 12; best epoch recorded	39,688 for 8-channel datasets; 39,194 in the UCI HAR full-pipeline count
Adapted TranAD	official TranAD model class; dynamic 6/8-channel adapter; sliding subwindows from length-128 HRA-SL windows	official AdamW loop; 5 epochs; patience 3; batch size 128; official commit 7ffb98d0	train-side calibration windows; same downstream probe; adaptation failures and reruns retained	7,208 for 8-channel datasets; 4,434 for UCI HAR

Table S6. Air Quality diagnostic summaries. These diagnostics explain why Air Quality is the hardest base signal in the evaluation.

Diagnostic	Evidence
Chronological shift	Standardized mean differences exceed one in PT08.S4(NO2), NO2(GT), and NOx(GT).
Shared background variation	The first two principal components explain about 0.89 of test-set variance.
Cross-channel coupling	Mean absolute raw-channel correlation in the test block is 0.784, with maximum absolute correlation 0.980.
Hard local perturbations	Noise-floor, saturation, offset, and scale perturbations have the lowest HRA-attribution top-1 localization on Air Quality.

Evidence paths.

The tables above summarize the ridge-alpha sensitivity analysis, split-scan analysis, hierarchy/flat design sensitivity, adapted TranAD comparison, deep-baseline configuration summary, supervised shortlisting comparison, and Air Quality diagnostics reported in the reproducibility artifact.
